# “Teledentistry” using a mobile app (Telesmile) to improve oral health among the visually impaired and hearing-impaired populations in Saudi Arabia: a randomized controlled study

**DOI:** 10.3389/froh.2024.1496222

**Published:** 2024-12-04

**Authors:** Hytham N. Fageeh, Manawar A. Mansoor, Hammam I. Fageeh, Hina N. Abdul, Hamza Khan, Abdulrahman Akkam, Idris Muhaddili, Sultan Korairi, Ashok Kumar Bhati

**Affiliations:** ^1^ Department of Preventive Dental Sciences, College of Dentistry, Jazan University, Jazan, Saudi Arabia; ^2^ Department of Prosthetic Dental Sciences, College of Dentistry, Jazan University, Jazan, Saudi Arabia; ^3^ College of Dentistry, Jazan University, Jazan, Saudi Arabia

**Keywords:** oral health, teledentistry, visually impaired, hearing-impaired, oral hygiene

## Abstract

**Objective:**

To assess the efficacy of a “teledentistry” method using a mobile app (Telesmile) in enhancing knowledge of oral health conditions and oral hygiene practices among the blind and deaf populations in Jazan Province in Saudi Arabia.

**Methods:**

A randomized parallel design controlled study was conducted among 50 blind and 50 deaf subjects between the ages of 12–18 years, randomly chosen from blind and deaf schools. The participants were selected based on the inclusion and exclusion criteria. An innovative teledentistry platform named Telesmile, a mobile application for the Apple iOS App Store and Google Play Store, was developed. Multimedia Arabic dental sign language oral hygiene instructional videos were created and uploaded in the Telesmile mobile application under the deaf category. Similarly, oral hygiene instructions were audio recorded by experts and uploaded under the blind category in the Telesmile mobile application. Group I of the blind (*n* = 25) and deaf participants (*n* = 25) received regular oral hygiene instructions while Group II of the blind (*n* = 25) and deaf (*n* = 25) participants received the Telesmile mobile application intervention. The knowledge of the participants pertaining to oral health and oral hygiene practices was evaluated using a close-ended questionnaire comprising 14 questions at the initial visit (T0). Training sessions were conducted for all participants and the Telesmile mobile application was distributed among the participants in Group II. After 4 weeks (T1), the knowledge of the participants regarding oral health and hygiene practice was re-evaluated and compared between each group.

**Results:**

The chi-square test revealed that the marginal mean of the knowledge of the blind and deaf participants pertaining to oral health and oral hygiene practices was very poor at their initial visit (T0) and it significantly increased (*p* < 0.001) after 4 weeks (T1) of using the Telesmile mobile application.

**Conclusion:**

The Telesmile mobile application can significantly enhance oral hygiene knowledge among blind and deaf people. The audio technique was an effective tool to deliver oral health education which could result in improving the oral health status of blind participants. The video demonstrations were also effective in enhancing the oral health and oral hygiene knowledge of deaf individuals.

## 1 Introduction

The significance of appropriate daily oral health routines and oral hygiene services for everybody cannot be overstated (1). In recent years, demand has increased for a wider reach as dental professionals, especially specialists, might not be easily available due to the need to travel long distances, the lack of transport facilities, ignorance regarding oral health, geographical constraints to care, and cultural barriers (2, 3). Due to this inconsistency in oral healthcare delivery and accessibility between rural and urban areas, it is necessary to implement “teledentistry,” a new idea to provide dental treatment across distances (4). Similar to telemedicine, teledentistry could be delivered in the form of teleconsultation, tele-education, telemonitoring, and telesurgery through the following two major ways: “videoconferencing” where dentist and patients can communicate with one another over long distances and “store-and-forward” teledentistry where clinical information is collected, stored, and conveyed using telecommunications equipment by a dental professional. Thus, the stored information can be forwarded for consultation and treatment planning as required (5–7).

According to the American Dental Association (ADA), teledentistry also includes mobile health (m-health), comprising healthcare practices and educational aids using mobile communication such as mobile phones, tablets, and computers (8). The Telesmile application is a mobile application with a two-way communication platform between the patient and practitioner. In the Telesmile application, the “May I help you” icon is available where patients can write their chief complaint and personal details and can upload pictures of any existing complaints. Furthermore, these patients can be contacted on their telephone after their complaints are reviewed.

Smartphone-based mobile applications can become a powerful tool in improving oral health education due to their various advantages compared to traditional booklets and verbal instructions. The application can be used anytime and anywhere to access the information. The application has videos, animations, and interactive guides that make learning more interactive and engaging compared to traditional pamphlets or verbal instructions. These tools can help users understand complex dental care procedures and concepts more easily. Based on users’ data on oral health habits, age, diet, and risk factors, the application offers personalized advice, reminders, and preventive tips, improving the relevance and effectiveness of the education. The application provides consistent reminders that help users maintain good oral hygiene habits. Moreover, it has a wide range of educational materials to improve oral health and is available free of charge (9, 10).

The incidence of visual impairment and hearing impairment is increasing across the globe. Worldwide, there are 45 million people who are blind or visually impaired and most of them live in developing countries, including Saudi Arabia. Nearly 1 million people in the Kingdom of Saudi Arabia are visually impaired (11). It is reported that by 2050, nearly 2.5 billion people are projected to have some degree of hearing loss, and at least 700 million will require hearing rehabilitation with above 1 billion young adults at risk of permanent, avoidable hearing loss due to unsafe listening practices (12). According to the Ministry of Economy and Planning, the deaf population in Saudi Arabia exceeds 7,20,000 (13). Studies comparing the periodontal status and oral hygiene of blind children, teenagers, and elderly people with normal individuals have shown that the oral health of the sighted groups is better compared to the blind groups (14–16). Numerous research studies have been conducted showing that hearing-impaired individuals have poorer oral hygiene, a high prevalence of caries, and an unfulfilled need for treatment (17, 18).

In general, oral hygiene practice training is given using various visual materials considering the characteristics of people with disabilities (19). This typically includes the use of visual aids such as disclosing tablets and models. This kind of training is a single-time event and is organized in a traditional format; it is impracticable for building a correct oral health maintenance lifestyle in day-to-day life (20). However, there may be instances when demonstrations and visual aids are inappropriate, for instance, when the patient is blind or visually impaired (21, 22). Furthermore, the visual materials utilized in such training and learning programs have restrictions when engaging with and encouraging people with disabilities to learn tooth brushing patterns, and challenges in sustaining the effectiveness of the learning (23). Similarly, oral health status and oral hygiene practice are usually neglected among patients with hearing impairments either due to insufficient communication or due to societal misconceptions concerning the social standing of these individuals (24).

Moreover, there has been a phenomenal increase in smartphone users and a huge trade of mobile applications for multiple goals, including health and oral healthcare (25). Sadly, the majority of mobile apps aimed at attaining efficient communication depend on the misunderstanding that the majority of deaf or blind people are literate (26). Furthermore, a small fraction of literate deaf people have little health literacy and do not understand the precise terminology that health providers use (27). Digital media applications can offer numerous concrete ideas (movies, subtitles, and sign language) for the deaf and hard of hearing (28). Therefore, the current research study was conducted to determine the effectiveness of the use of teledentistry in oral health amongst visually impaired and hearing-impaired patients. The study was conducted to test the null hypothesis that there would be no difference in the knowledge pertaining to oral health and oral hygiene practices among the blind and deaf participants after using the Telesmile mobile application.

## 2 Material and method

### 2.1 Study population

**Figure 1:**
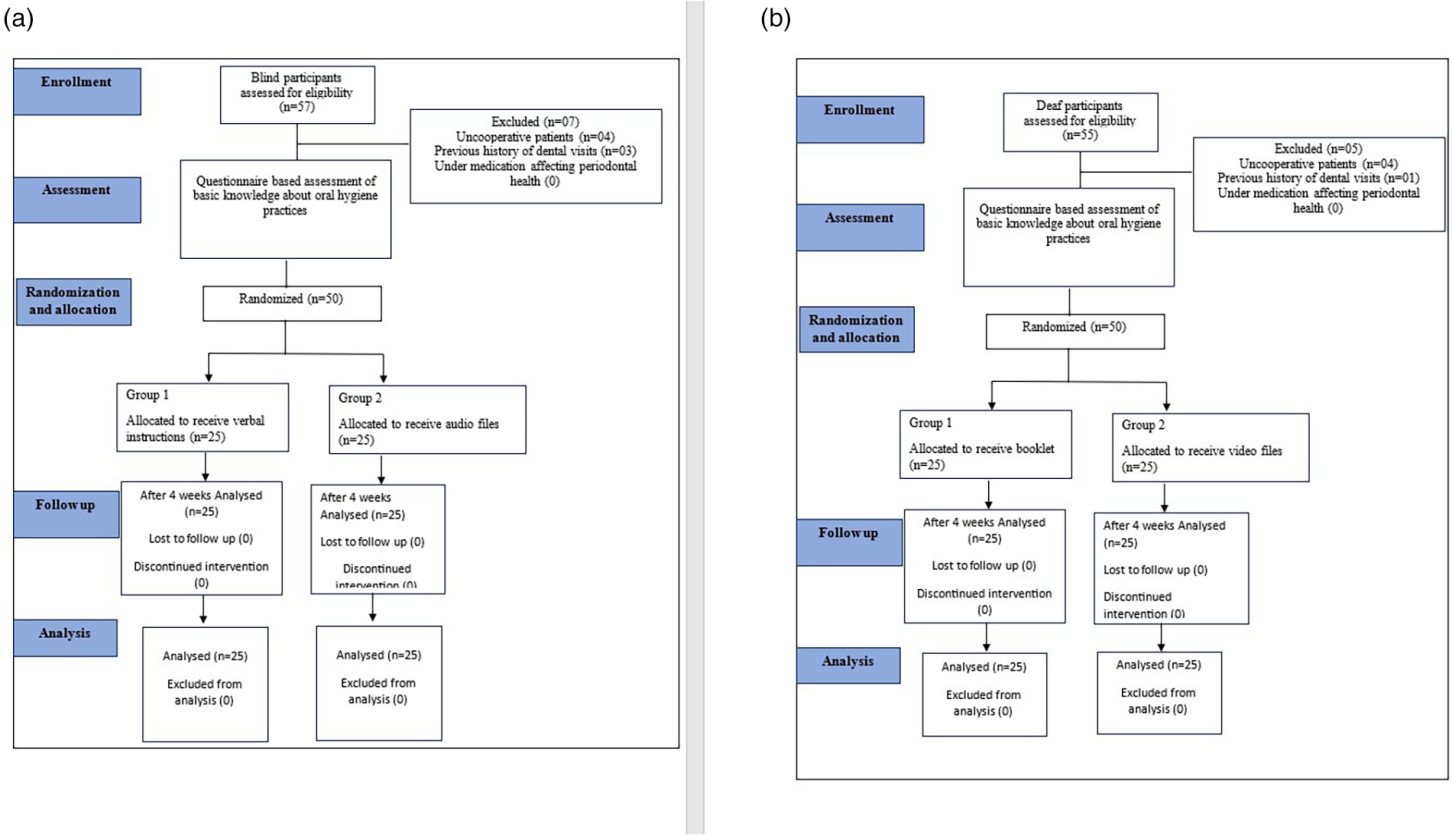
**(a,b)** CONSORT study flow chart for the blind and deaf participants.

The study population comprised 50 blind and 50 deaf participants (age 12–18 years), randomly chosen from the schools for the blind and schools for the deaf in Jazan Province, Saudi Arabia. The participants were selected based on the inclusion and exclusion criteria. The inclusion criteria were as follows: willingness to participate in the study; no professional prophylaxis over the past 3 months; and no orthodontic banding or removable prosthesis. The exclusion criteria were as follows: uncooperative patients; participants receiving medications that affected their gingival or periodontal health; and any previous history of dental visits. The Consolidated Standards of Reporting Trials (CONSORT) guidelines on reporting randomized controlled trials have been followed. Figure 1 shows the CONSORT flow chart with the patient flow and intervention.

### 2.2 Informed consent and ethical clearance

The goal of the study was described to the participants, and written informed consent was collected from the parents or caregiver of the blind and deaf participants. The study was conducted according to the guidelines of the Helsinki Declaration, and ethical clearance was obtained from the Standing Committee for Scientific Research Ethics at Jazan University (Ref: REC-43/06/120).

### 2.3 Fabrication of audio and video files

An Arabic oral hygiene instructional booklet was used to provide regular oral hygiene instructions in Group I to both the blind and deaf participants (29). The booklet was communicated by Arabic sign language experts at the deaf school in Jazan, Saudi Arabia. A group comprising four dental interns was trained to give these instructions by the sign language experts. A total of 10 videos (MP4 files) were developed using the identical content present in the booklet. International guidelines were followed to ensure that the backgrounds were dark enough for a clear interpretation of the sign language. All videos had running subtitles in Arabic for better clarity. A total of 10 audio clips (MP3 files) were recorded related to the oral hygiene instructions for the blind participants.

### 2.4 Development of the mobile application

**Figure 2:**
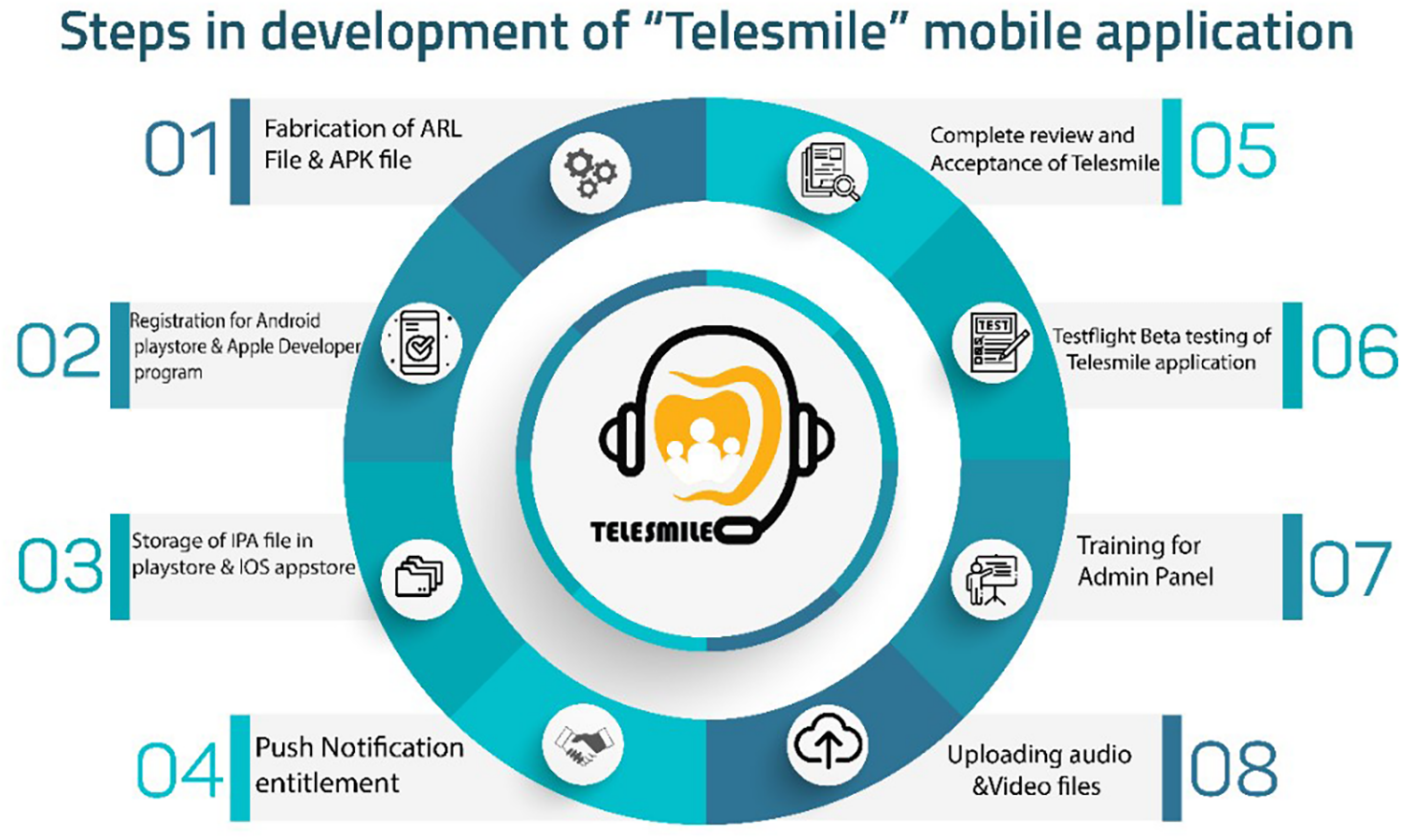
Steps in the development of the Telesmile mobile application for Android and iOS.

**Figure 3:**
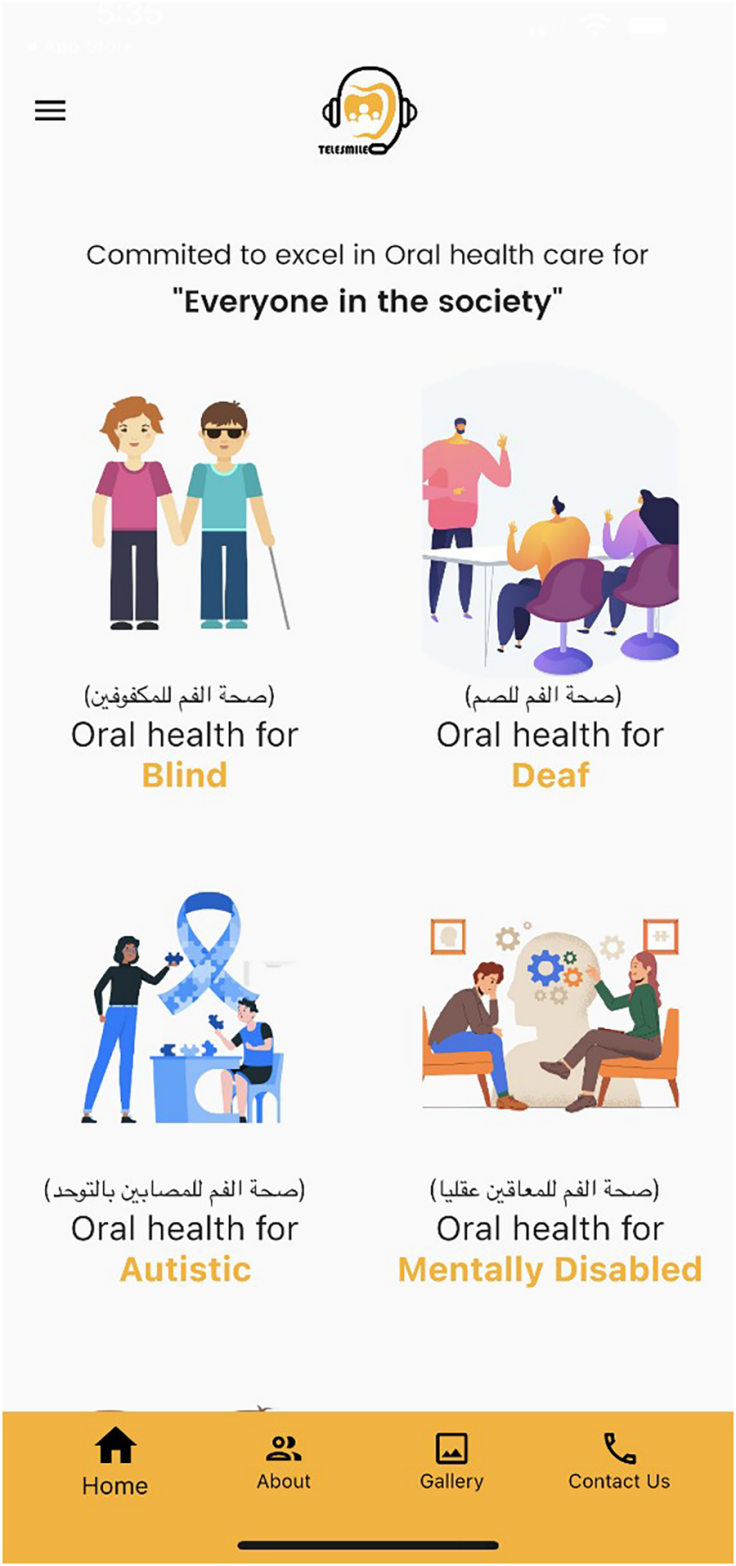
Front screen of the Telesmile mobile application for both blind and deaf participants.

**Figure 4:**
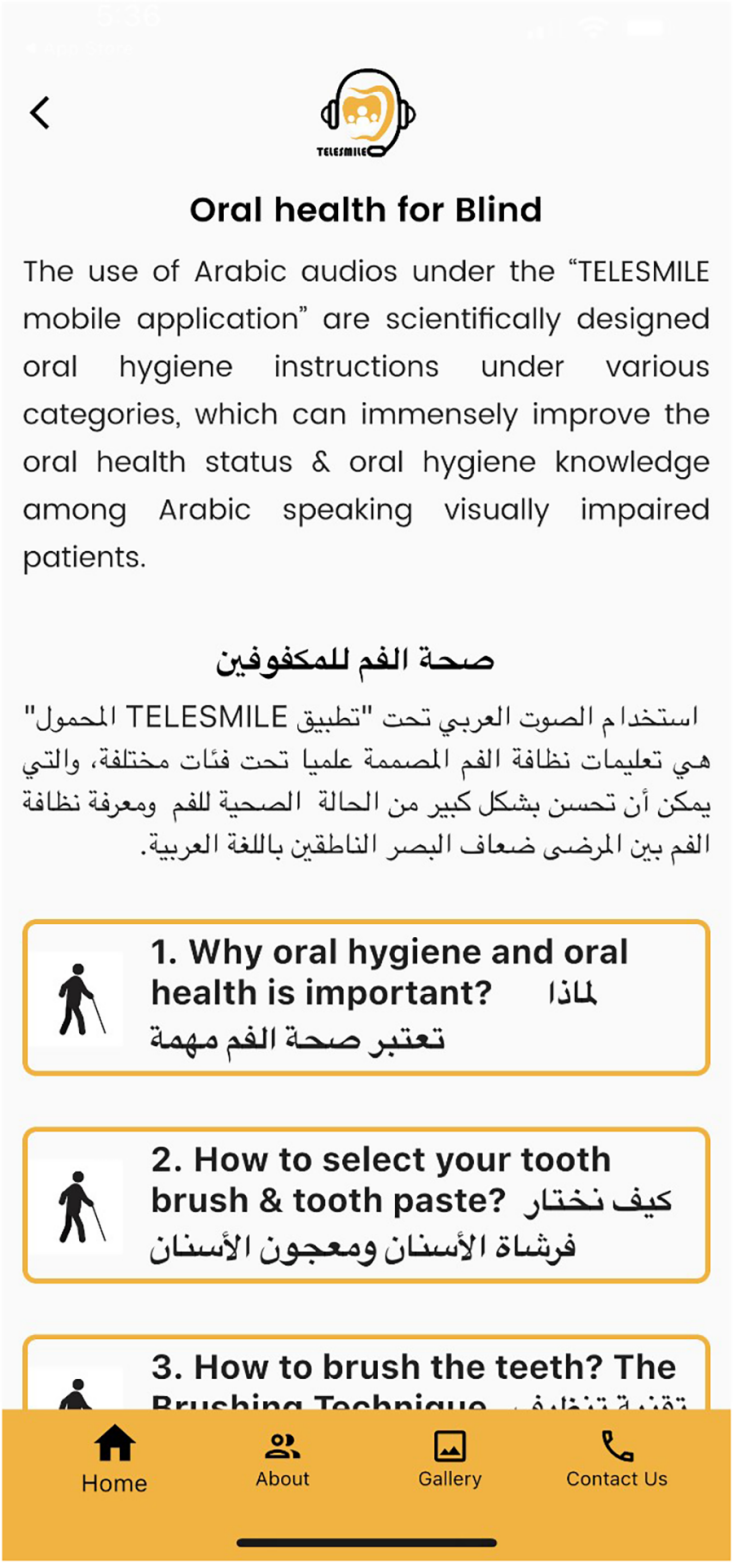
Screen showing the various oral hygiene instructions for the blind participants.

**Figure 5:**
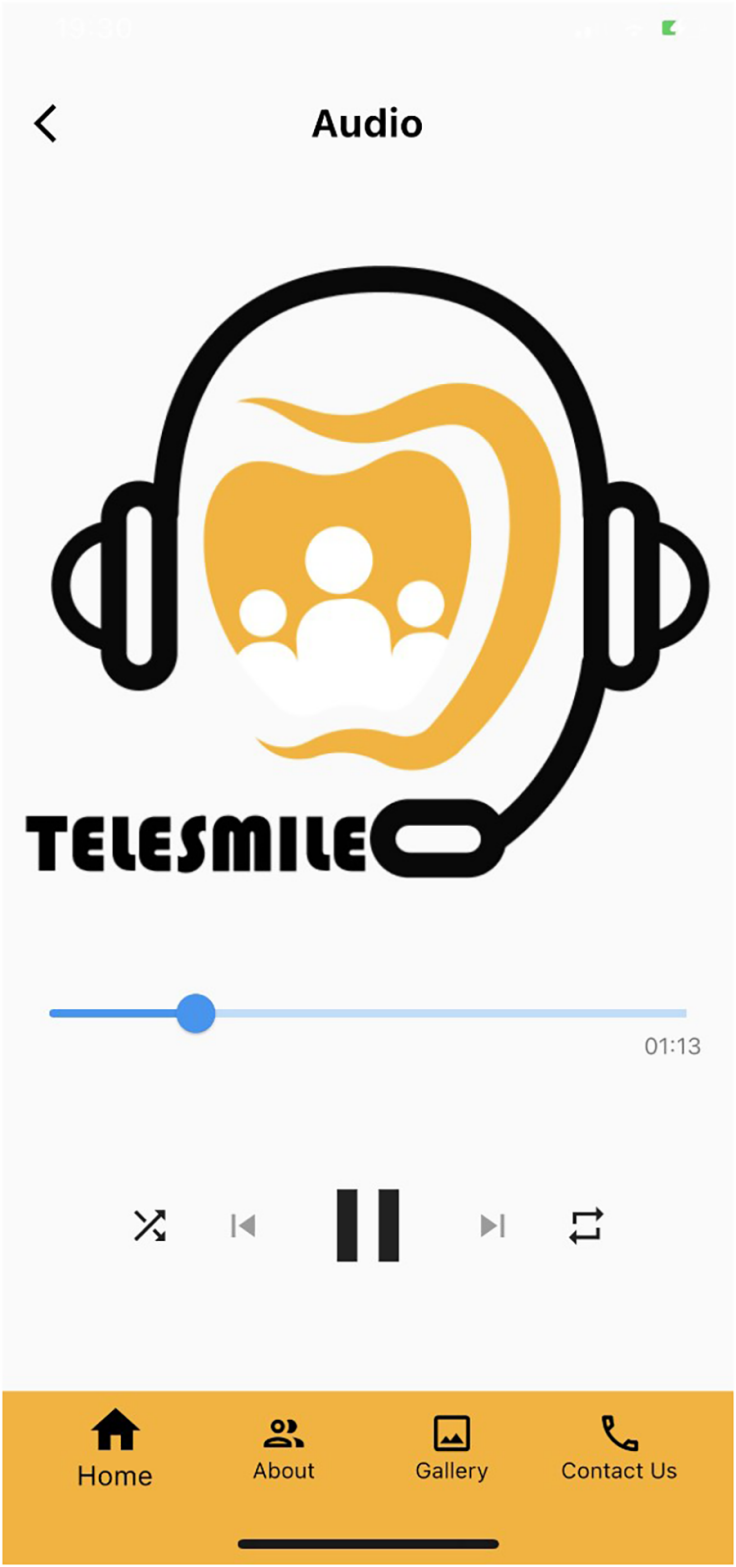
Screen showing the audio clips for the blind participants to listen to the oral hygiene instructions.

**Figure 6:**
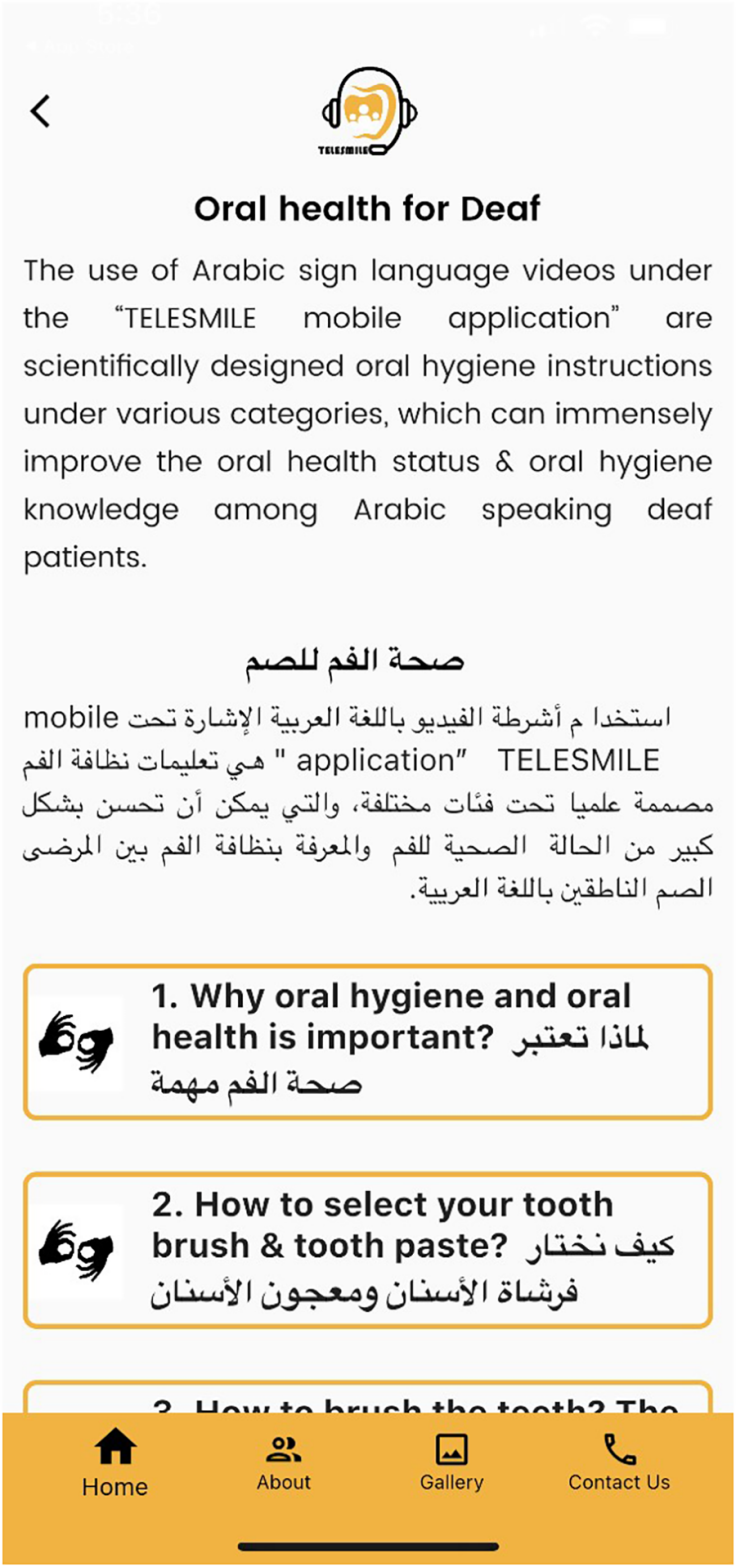
Screen showing various oral hygiene instructions for the deaf participants.

**Figure 7:**
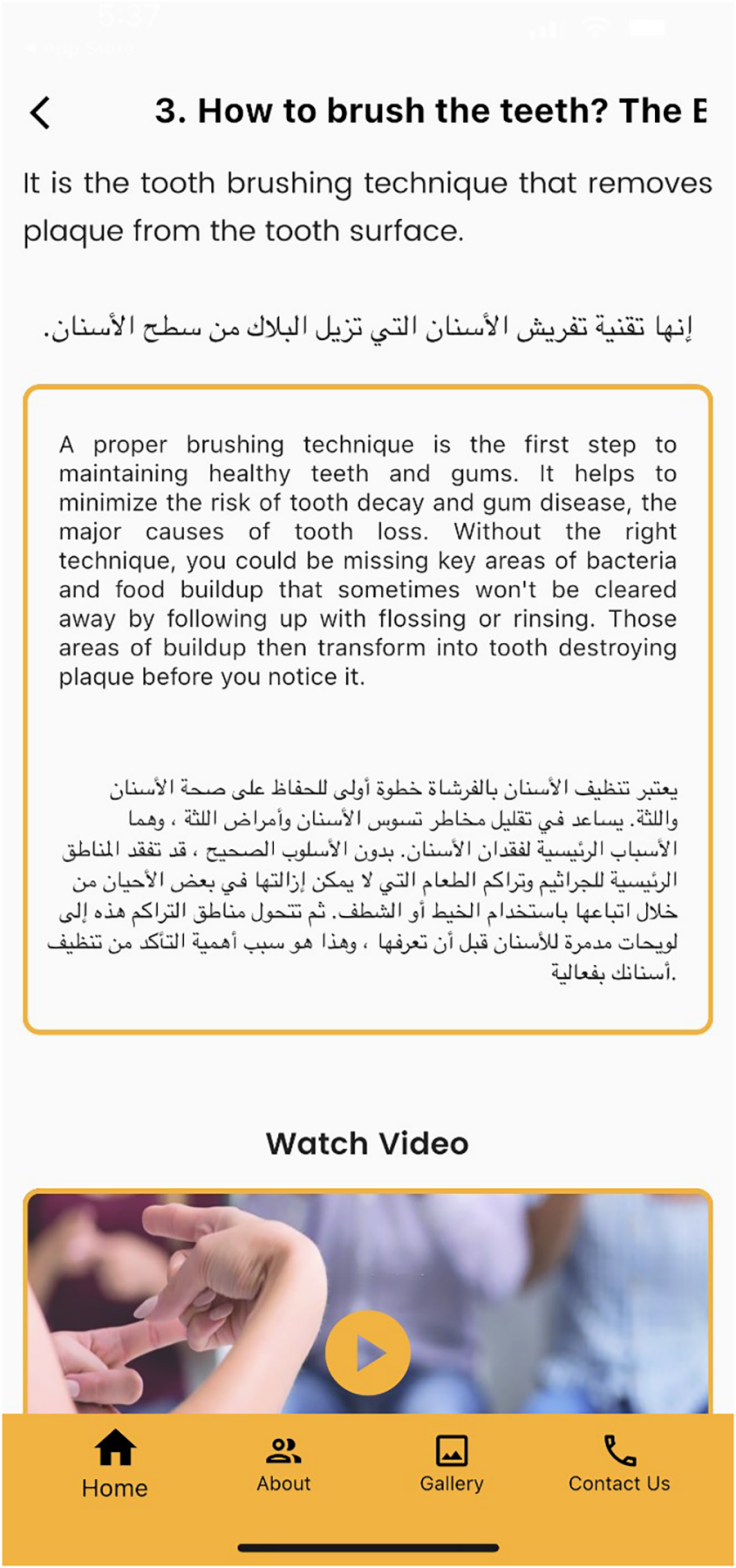
Screen showing various video clips related to oral hygiene instructions for the deaf participants.

**Figure 8:**
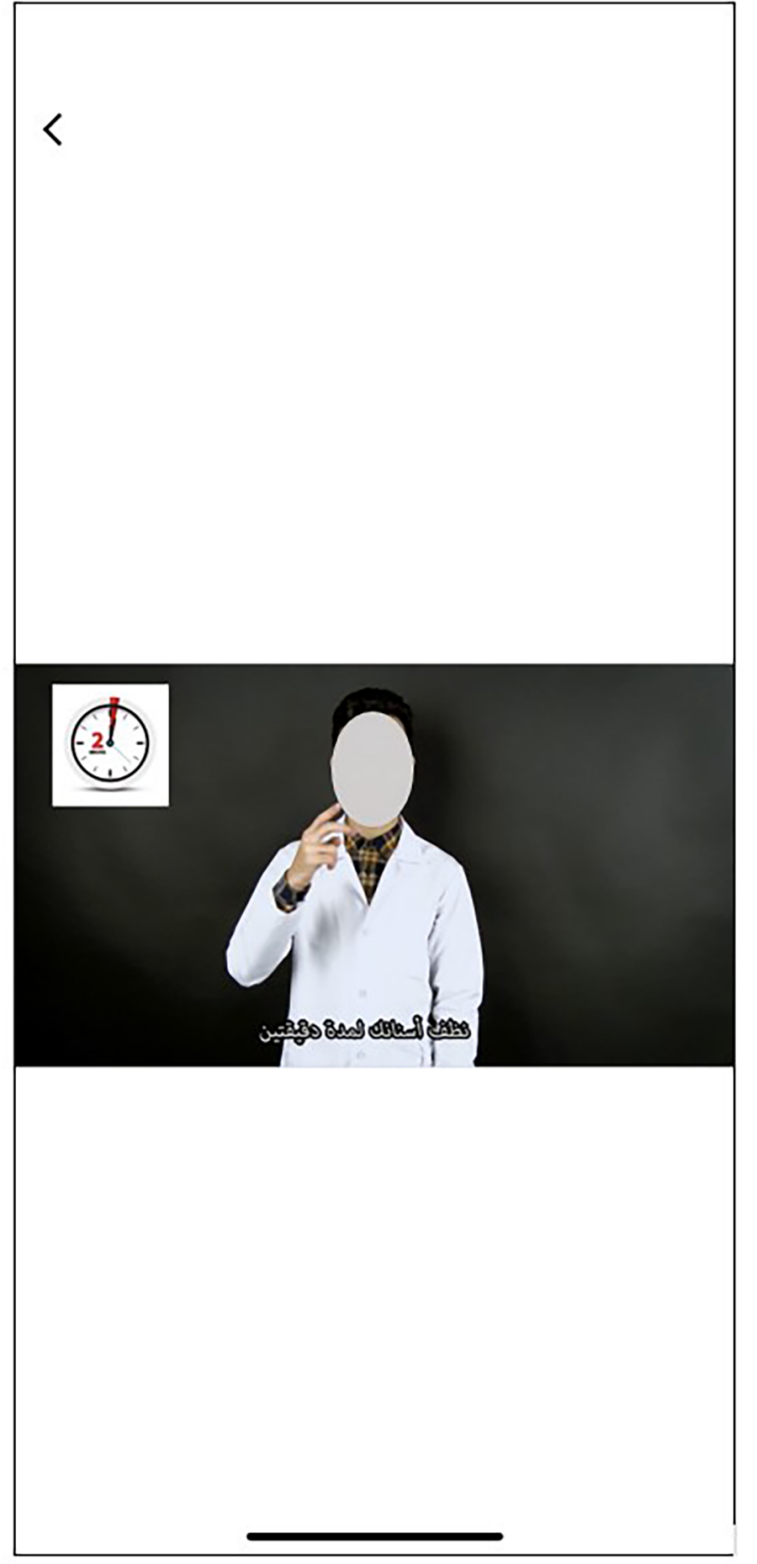
Screen showing an Arabic sign language video clip for the deaf participants to watch the oral hygiene instructions.

Concurrently, a software programmer (Studyleague IT solutions, India) developed a first draft of the Telesmile mobile application for Android and iPhone mobile devices using a Corona (Corona Labs, CA, USA) framework, which was evaluated by the team in terms of colors, contrast, usability, performance, and vignettes. After completing the ARL (Application Recognition Library) file, the APK (Android Package Kit) file was registered in the Google Play Store with the software programming codes and assets. The mobile application content and design were evaluated by experts. After its verification, a counterpart of the APK file i.e., the IPA (iOS Package App Store) file was uploaded to the iOS App Store for use on iPhones. Push Notification entitlement was rectified with the APS (Apple Push Notification Service) environment key several times to make sure that the application was configured correctly and to ensure the acceptance of the Telesmile application in the Apple App Store. After the complete review and acceptance of the Telesmile mobile application, beta testing was conducted to evaluate the mobile application’s performance in real time and to uncover any bugs or issues before releasing the mobile application to the target audience (Figure 2). At the same time, investigators were trained as an admin panel to control and upload the audio and video multimedia files in the mobile application. Once the Telesmile mobile application was launched in the Apple App Store and Google Play Store, the 10 audio files (MP3 files) for the blind category and 10 video files (MP4 files) for the deaf category were uploaded successfully and were ready to be used by the target audience (Figures 3–8).

### 2.5 Questionnaire for assessment of knowledge

A close-ended questionnaire containing 14 questions for the blind and deaf participants was designed and translated into Arabic to evaluate their knowledge, attitude, and practices pertaining to oral health and hygiene. The designed questionnaire and translation were already validated as the questionnaire has been used in published studies (30). A pilot study was performed to test and validate the questionnaire (Cronbach's alpha = 0.91). The participants answered the questionnaire pre- and post-viewing the media in the mobile application. All the questions had multiple choice answers to which a correct answer was scored “1” whereas a wrong answer was scored “0,” thereby allowing the calculation of a total knowledge score for each participant.

### 2.6 Study protocol

This study was a randomized controlled parallel design study conducted from February 2022 to May 2022 at the College of Dentistry, Jazan University, Saudi Arabia. In total, 50 blind and 50 deaf participants were chosen from schools for the blind and schools for the deaf in Jazan Province, Saudi Arabia, were included in the study. The sample size for each group was as per a previously published study determining the effectiveness of oral hygiene instructions in sign language among hearing-impaired adults in Saudi Arabia (30). Each participant category was subdivided into two groups, Group I (did not receive the intervention) and Group II (received the intervention), with 25 individuals in each group. All the groups received the questionnaire in school. The questionnaire-based assessment of their basic knowledge of oral health hygiene practices was done prior to the intervention. The examiner was unaware of any allotment or any information regarding the participants associated with the study and was blinded at all times. A trial protocol was followed before the start of the study. An expert from the school for the blind or school for the deaf was with the responder, who ensured that the participant had completely understood the questions. After finishing, the link for the mobile application was sent to the Group II blind and deaf participants, however, the participants in Group I continued with the verbal instructions for the blind participants and booklet instructions for the deaf participants. The Group II participants were requested to listen to the audio files (blind participants) or watch video files (deaf participants) on oral hygiene practices in the Telesmile mobile application once a day for 4 weeks. The deaf participants were given similar instructions to read the booklet once a day for 4 weeks. Similarly, the blind participants were also given the verbal instructions every day for a period of 4 weeks. The participants in Group II were asked to use the mobile application in the presence of their caregivers who made sure that the participants used the mobile application every day for 4 weeks. After the 4 weeks, a similar questionnaire-based knowledge evaluation was performed to determine the efficacy of the teledentistry method using a mobile app (Telesmile) in enhancing the knowledge of oral health conditions and oral hygiene practices among the blind and deaf participants.

### 2.7 Randomization

In total, 50 blind and 50 deaf subjects were randomly assigned to Groups I and II using an envelope containing their group number. Group I participants did not receive the intervention whereas Group II received the intervention as described above in the study protocol. A biostatistician prepared the envelope using random computer-generated number sequences to conceal the sequence generation and allocation. The envelopes were randomly distributed to the patients by an assistant unconnected to the study who was unaware of its contents. The participants were assigned into two equal subgroups with an allocation ratio of 1:1 (*n* = 25).

### 2.8 Statistical analysis

The collected data were analyzed using the Statistical Package for the Social Sciences (SPSS) for Windows, version 20.0 (SPSS Inc., Chicago, IL, USA). Chi-square tests were used to assess the knowledge regarding oral health and oral hygiene practice after the use of the Telesmile mobile application among the blind and hearing-impaired participants, respectively. The level of statistical significance was set at 5%.

## 3 Results

In total, 50 blind and 50 deaf participants with a mean age of 15.6 years for the deaf participants and 15.7 years for the blind participants were included in the study. Figures 1a,b show the study flow chart with the participant flow and intervention. The current study was conducted to evaluate the efficacy of the Telesmile mobile application in improving the knowledge of deaf and blind participants pertaining to oral health and oral hygiene practice. The trial ended on completion of the study and no harm was done to the participants during the study. A total of 14 closed-ended questions were used in the questionnaire with a score of “1” for the correct answer and a score of “0” for the wrong answer (Table 1).

**Table 1:** Questionnaire used to assess the knowledge regarding oral hygiene practice among blind and deaf subjects.

S. No.	Questions	Multiple choice options	Response
1.	How many times should you brush your teeth in a day?	a. Do not brush the teeth	
		b. One time per day	
		c. Two times per day	
		d. Once or two times a week	
2.	What type of bristles in the toothbrush should you use?	a. Toothbrush with any type of tooth bristles (I never noticed)	
		b. Toothbrush with soft bristles	
		c. Toothbrush with medium-hard bristles	
		d. Toothbrush with hard bristles	
3.	What type of toothpaste should you use to prevent tooth decay?	a. Fluoride tooth paste	
		b. Desensitizing toothpaste	
		c. Anti-plaque toothpaste	
		d. Any type of toothpaste	
4.	What direction should you use the toothbrush while brushing your teeth with a toothbrush?	a. Keep the toothbrush bristles parallel to the tooth surfaces	
		b. Keep the toothbrush bristles perpendicular to the tooth surfaces	
		c. Keep the toothbrush bristles at 45 degrees to the tooth surface	
		d. Brush teeth in all possible directions	
5.	How often do should you replace your toothbrush?	a. After 3 month	
		b. After 6 months	
		c. After 1 year	
		d. I don't know	
6.	How many times should you use interdental brush?	a. Once per day	
		b. Two times per day	
		c. Once per week	
		d. I don't know (I have not heard about this type of toothbrush)	
7.	How many times should you floss your teeth with dental floss?	a. Once per day	
		b. Two times per day	
		c. Once per week	
		d. Once per month	
8.	What type of mouthwash should you use to reduce the bacteria and gingivitis?	a. Cosmetic mouthwash	
		b. Fluoride mouthwash	
		c. Therapeutic mouthwash	
		d. Any type of mouthwash	
9.	How often should you visit your dentist for a regular general dental check-up and oral prophylaxis?	a. Once in 3 months	
		b. Once in 6 months	
		c. Once in a year	
		d. Don't visit any dentist unless you have any dental problem	
10.	What type of diet will keep your gums and teeth healthy?	a. Sugary foods with every meal such as cake, pastry, and ice creams	
		b. Soft drinks with every meal such as Coca-Cola or Pepsi	
		c. Fruit juices after every meal	
		d. Fibrous fruits after every meal such as apples, carrots, and salad	
11.	What is the reason for teeth sensitivity after a routine scaling procedure is conducted by a dentist?	a. Dentist removes the part of the tooth during the scaling procedure	
		b. Dentist removes the gum from the tooth surface during the scaling procedure	
		c. The tooth becomes weak and breaks by itself after the scaling procedure	
		d. Unwanted deposits are removed from root surfaces, temporarily leaving teeth sensitive to temperature changes	
12.	After using a mouthwash, how much time should you wait before drinking water or eating food?	a. 5 min	
		b. Half an hour	
		c. 2 h	
		d. I don't know	
13.	While using a mouthwash, how long should you swish the mouthwash around your teeth?	a. Take it inside the mouth and spit immediately to avoid swallowing	
		b. 30–60 s	
		c. 5 min	
		d. I don't know	
14.	What type of toothbrush will most frequently damage the gums and cause gum diseases?	a. Toothbrush with soft bristles	
		b. Toothbrush with medium bristles	
		c. Toothbrush with hard bristles	
		d. Electronic toothbrush	

A comparison of the correct responses to all 14 questions before and after the intervention using the chi-square test was made between the blind participants who received the intervention with the Telesmile mobile application and the blind participants who received verbal instructions, as well as the deaf participants who received the intervention with the Telesmile application and deaf participants who received the booklet instructions. A correct response to all 14 questions after the intervention with the Telesmile mobile application was significantly more likely (*p* < 0.001) among both the blind and deaf participants compared to the blind participants who received verbal instructions and the deaf participants who received booklet instructions, respectively (Table 2).

**Table 2:** Questionnaire-based knowledge assessment regarding oral health and oral hygiene practices before and after using the Telesmile mobile application among blind and deaf participants.

Questions in the questionnaire	Blind subjects				Deaf subjects				Chi-square value	*p*-value
	Verbal instructions		Telesmile mobile application		Booklet instructions		Telesmile mobile application			
	Before	After	Before	After	Before	After	Before	After		
Q1	6 (24%)	7 (28%)	12 (48%)	22 (88%)	4 (16%)	6 (24%)	12 (48%)	23 (92%)	27.239	<0.001
Q2	6 (24%)	6 (24%)	6 (24%)	19 (76%)	6 (24%)	10 (40%)	6 (24%)	23 (92%)	29.903	<0.001
Q3	2 (8%)	4 (16%)	6 (24%)	20 (80%)	2 (8%)	4 (16%)	10 (40%)	21 (84%)	20.502	<0.001
Q4	1 (4%)	4 (16%)	4 (16%)	17 (68%)	4 (16%)	4 (16%)	3 (12%)	19 (76%)	34.415	<0.001
Q5	11 (44%)	9 (36%)	10 (40%)	21 (84%)	10 (40%)	10 (40%)	12 (48%)	22 (88%)	25.066	<0.003
Q6	1 (4%)	0 (0%)	3 (12%)	16 (64%)	2 (8%)	6 (24%)	11 (44%)	14 (56%)	14.148	<0.028
Q7	1 (4%)	5 (20%)	7 (28%)	18 (72%)	2 (8%)	5 (20%)	1 (4%)	4 (16%)	19.350	<0.001
Q8	2 (8%)	9 (36%)	4 (16%)	17 (68%)	7 (28%)	8 (32%)	3 (12%)	23 (92%)	21.116	<0.001
Q9	2 (8%)	7 (28%)	4 (16%)	20 (80%)	3 (12%)	4 (16%)	2 (8%)	20 (80%)	17.905	<0.029
Q10	16 (64%)	16 (64%)	18 (72%)	23 (92%)	9 (36%)	10 (40%)	8 (32%)	18 (72%)	27.659	<0.001
Q11	12 (48%)	5 (20%)	15 (60%)	21 (84%)	10 (40%)	11 (44%)	5 (20%)	15 (60%)	30 498	<0.001
Q12	8 (32%)	11 (44%)	3 (12%)	19 (76%)	6 (24%)	5 (20%)	6 (24%)	12 (48%)	23.106	<0.001
Q13	9 (36%)	6 (24%)	12 (48%)	18 (72%)	8 (32%)	5 (20%)	5 (20%)	16 (64%)	25.709	<0.001
Q14	11 (44%)	12 (48%)	18 (72%)	20 (80%)	6 (24%)	8 (32%)	8 (32%)	15 (60%)	21.909	<0.002

Furthermore, the 50 hearing-impaired subjects and 50 visually impaired participants demonstrated inadequate knowledge pertaining to the frequency of brushing required in a day, the type of toothbrush bristles to use, the type of toothpaste to use to prevent tooth decay, and the appropriate tooth brushing techniques to use. Before providing oral hygiene instructions, only 6 (24%) patients in Group I and 12 (48%) in Group II provided correct answers for the number of times they should brush their teeth every day (Q1) among the blind participants; these numbers increased to 7 (28%) and 22 (88%) after 4 weeks of verbal instructions and the intervention with the Telesmile mobile application, respectively. Among the deaf participants, only 4 (16%) patients in Group I and 12 (48%) in Group II provided correct answers for the number of times they should brush their teeth every day (Q1); these numbers increased to 6 (24%) and 23 (92%) after 4 weeks of booklet instructions and the intervention with the Telesmile mobile application, respectively (Supplementary Figure S1).

Similarly, substantial increases in the number of blind and deaf participants showing ample knowledge pertaining to the type of toothbrush bristles to use, type of toothpaste to use to prevent tooth decay, appropriate tooth brushing techniques, and when to replace a used toothbrush (Q2, Q3, Q4, and Q5) were noted in Group II (Table 2; Supplementary Figure S1–S5).

The majority of both the blind and deaf participants showed a lack of knowledge about the use of interdental brushes and dental floss (Table 2, Q6 and Q7). Regarding the provision of booklet instructions, only a mild improvement was observed in the deaf participants in Group I; however, the proportion of patients who showed knowledge pertaining to the use of interdental brushes and dental floss increased to 56% and 16% among the deaf participants and 64% and 72% among the blind participants, respectively, in Group II, 4 weeks after receiving instructions from the Arabic sign language videos and verbal instructions in the Telesmile mobile application, respectively (Supplementary Figure S6, S7).

Only a few of the participants, 8% of the blind participants and 28% of the deaf participants, in Group I answered correctly pertaining to the use of mouthwash (Table 2, Q8). After 4 weeks of using the Telesmile mobile application usage, the proportion increased to 68% and 92% in Group II among the blind and deaf participants, respectively (Supplementary Figure S8).

As seen in Table 2 and Supplementary Figure S9, only 8% of the blind participants in Group 1% and 16% in Group II showed some knowledge pertaining to routine dental check-ups (Q9, Supplementary Figure S9).

This was 12% for deaf participants in Group I and 8% for those in Group II. After the verbal instructions, the proportion of participants who provided the correct answer to this question increased to only 28% in Group I; however, it significantly increased to 80% in Group II among both the blind and deaf participants. Both groups showed sufficient knowledge pertaining to the type of diet that is essential for maintaining sufficient periodontal health (Q10, Supplementary Figure S10).

With regard to knowledge pertaining to tooth sensitivity after oral prophylaxis (Q11), the use of mouthwash (Q12 and Q13), and the type of toothbrush that is recommended to prevent gum damage (Q14), a substantial improvement was seen among both blind and deaf participants in Group II after the provision of oral hygiene instructions using the Telesmile mobile application (Table 2, Supplementary Figures S11–S14).

## 4 Discussion

The null hypothesis was that there would be no difference between the use of the mobile application and the verbal or booklet instructions. However, there was a significant improvement in the knowledge of the subjects after the usage of the Telesmile mobile application. A low *p*-value (≤0.05) in the present study suggests strong evidence against the null hypothesis. Thus, the null hypothesis was rejected. This study used a software-programmed mobile application, Telesmile, which included 10 Arabic audio files and 10 Arabic sign language video clips accompanied by subtitles that were related to oral health and hygiene instructions (Figures 3, 5). Previous literature has highlighted the poor oral and periodontal health and lack of education related to oral hygiene among blind and deaf students (31–35). Considering the scarcity of blind- and deaf-specific educational tools for oral hygiene instructions, the success of electronic educational programs for blind and deaf students (36), and the scarcity of mobile applications for teaching specific oral health and hygiene instructions to blind and deaf students; the design of a mobile application software for teaching oral health and oral hygiene to the blind and deaf populations was considered in this study. Gentry et al. studied the use of multimedia programs for reading comprehension among deaf people in Louisiana and Texas and highlighted the benefit of using electronic education, especially multimedia education, in the education of deaf people (37).

This study was conducted among participants aged 12–18 years from schools for the blind and schools for the deaf who could use the mobile application. A special feature programmed in the Telesmile mobile application for the blind population was its autoplay system, i.e., if the user opens the application and waits for 10 s, it automatically plays the 10 audio clips related to oral hygiene one by one without any scrolling (Figure 4). This helpful feature was explained to all the blind participants and their caregivers for better use of the Telesmile mobile application. A maximum of 10 videos were considered suitable for both categories, as additional videos could undermine the convenience of the app and thus the communication between the dentist and the deaf or blind patient, which may delay the dental appointment. The first instruction was about the importance of oral hygiene and oral health. The second instruction was about how to select a toothbrush and toothpaste. The third instruction related to the correct brushing technique for oral hygiene. The fourth instruction was about brushing accessories such as interdental brushes. The fifth instruction was about how to use dental floss. The sixth instruction was about how to use mouthwash for good oral health. The seventh instruction was about a healthy diet for healthy teeth and gums. The eighth, ninth, and tenth instructions were about the post-extraction guidelines, tooth sensitivity, and oral hygiene instructions for children, respectively.

The outcome of the study showed a remarkable improvement in the knowledge pertaining to oral health and oral hygiene practice among the blind and deaf participants after using the Telesmile mobile application. This can be attributed to the fact that in the present study, while developing the audio and video clips, the basic concepts were incorporated using brief, easy-to-understand, and clear sentences. Understanding long sentences is difficult for both blind and deaf students. The slow-talking of the presenter and the use of short easy-to-understand sentences in the audio files for the blind participants and video files for the deaf participants made the Telesmile mobile application more user-friendly in terms of educating them about oral health and oral hygiene maintenance. In the clips that required images for deeper understanding, images appropriate to the topic were positioned in the top right corner of the film window using Adobe Premiere Pro CS3 software. Educational images or animations assist students in the learning process. The clarity of hand and lip movements in the sign language videos was very important (Figure 7) as it allows deaf students to focus on the hand and lip movements in the videos. Accordingly, we sought to develop high-quality videos in a brief format with a view to prevent users from becoming tired.

The study showed improvement in knowledge about oral health and oral health practices with the use of the Telesmile application. However, further studies with a larger sample size that assess clinical parameters such as plaque index and bleeding on probing after the use of Telesmile application should be conducted.

## 5 Conclusion

The Telesmile mobile application can tremendously enhance the oral health and oral hygiene practice knowledge of blind (visually impaired) and deaf (hearing-impaired) individuals. The audio technique could be an efficient method of providing oral health education and improving the oral health status of visually impaired children. The video demonstrations could be an effective tool to improve the oral health and oral hygiene knowledge of deaf patients.

## Data Availability

The original contributions presented in the study are included in the article/Supplementary Material, further inquiries can be directed to the corresponding author.
