# Supplementary material for: “Teledentistry” using a mobile app (Telesmile) to improve oral health among the visually impaired and hearing-impaired populations in Saudi Arabia: a randomized controlled study
Source: Front Oral Health. 2024 Dec 4;5:1496222. doi: 10.3389/froh.2024.1496222 (PMC11652368; doi:10.3389/froh.2024.1496222)
Supplement: Supplementary file 1 [file Table1.docx]

Supplementary Material

**Supplementary Figure 1:** Knowledge assessment among blind and deaf subjects before and after using telesmile mobile application for question 1.

**Supplementary Figure 2:** Knowledge assessment among blind and deaf subjects before and after using telesmile mobile application for question 2

**Supplementary Figure 3:** Knowledge assessment among blind and deaf subjects before and after using telesmile mobile application for question 3

**Supplementary Figure 4:** Knowledge assessment among blind and deaf subjects before and after using telesmile mobile application for question 4

**Supplementary Figure 5:** Knowledge assessment among blind and deaf subjects before and after using telesmile mobile application for question 5

**Supplementary Figure 6:** Knowledge assessment among blind and deaf subjects before and after using telesmile mobile application for question 6

**Supplementary Figure 7**: Knowledge assessment among blind and deaf subjects before and after using telesmile mobile application for question 7

**Supplementary Figure 8:** Knowledge assessment among blind and deaf subjects before and after using telesmile mobile application for question 8


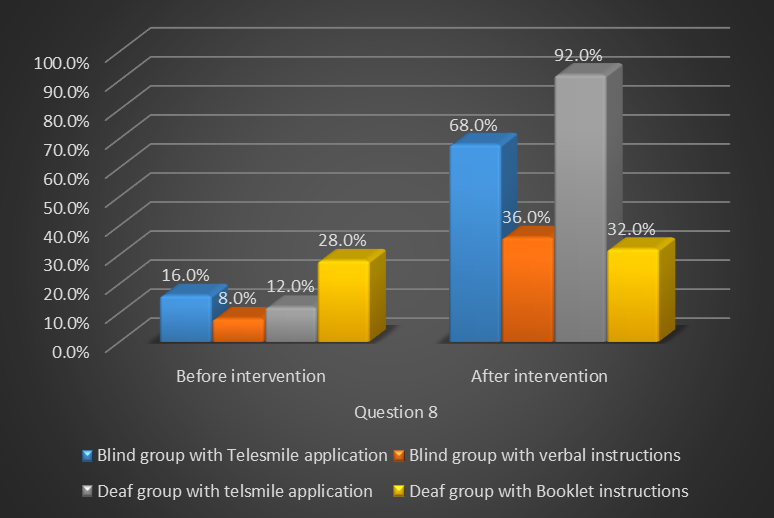


**Supplementary Figure 9:** Knowledge assessment among blind and deaf subjects before and after using telesmile mobile application for question 9

**Supplementary Figure 10:** Knowledge assessment among blind and deaf subjects before and after using telesmile mobile application for question 10

**Supplementary Figure 11:** Knowledge assessment among blind and deaf subjects before and after using telesmile mobile application for question 11

**Supplementary Figure 12:** Knowledge assessment among blind and deaf subjects before and after using telesmile mobile application for question 12

**Supplementary Figure 13:** Knowledge assessment among blind and deaf subjects before and after using telesmile mobile application for question 13

**Supplementary Figure 14:** Knowledge assessment among blind and deaf subjects before and after using telesmile mobile application for question 14
